# Vaginal cuff recurrence after radical cystectomy: an under - studied site of bladder cancer relapse

**DOI:** 10.1590/S1677-5538.IBJU.2017.0376

**Published:** 2018

**Authors:** Fabio Zattoni, Alessandro Morlacco, Avinash Nehra, Igor Frank, Stephen A. Boorjian, Prabin Thapa, R. Jeffrey Karnes

**Affiliations:** 1 Department of Urology, Mayo Clinic, Rochester, Minnesota, USA; 2 Clinica Urologica, Dipartimento di scienze Chirurgiche, Oncologiche e Gastroenterologiche, Azienda Ospedaliero - Universitaria di Padova, Padova, Italy; 3 Health Sciences Research, Mayo Clinic, Rochester, MN, USA

**Keywords:** Urinary Bladder Neoplasms, Female, Neoplasm Metastasis, Recurrence

## Abstract

**Introduction:**

Vaginal cuff recurrence of tumor following radical cystectomy is a rare site of disease recurrence, however it has never been specifically studied. The aim of the study is to evaluate incidence, risk factors, and long-term oncologic outcomes of vaginal cuff recur- rence in a cohort of female patients treated with radical cystectomy for invasive urothelial carcinoma of the bladder.

**Materials and Methods:**

From 1985 to 2012, a prospectively maintained institutional blad- der cancer registry was queried for vaginal cuff recurrence post radical cystectomy. Over- all mortality and cancer-specific mortality were reported using the Kaplan-Meier method for patients with vaginal cuff recurrence, recurrence at another local or distant site, and those without evidence of recurrence. Comparisons were performed using the log-rank test. Cox proportional hazards regression model was performed to assess predictors of vaginal cuff recurrence.

**Results:**

From 469 women treated with radical cystectomy for bladder cancer, 34 patients (7.3%) developed vaginal cuff recurrence, 130 patients (27.7%) had recurrence involving ei- ther a local or distant site, and 305 patients (65%) had no evidence of recurrence. The 5-year overall mortality-free survival rate was 32.4% for vaginal cuff recurrence, but 25.0% for other sites of recurrence. Cancer-specific mortality-free survival rate was 32.4% for vaginal cuff recurrence, and 30.3% for the other sites of recurrence. Multivariate Cox proportional hazards regression analysis demonstrated that the presence of tumor in posterior location at radical cystectomy (Hazard Ratio [HR], 0.353 [95% CI, 0.159-0.783]) and anterior vaginec- tomy, compared to no vaginectomy (HR, 2.595 [95% CI, 1.077-6.249]) were independently associated with vaginal cuff recurrence.

**Conclusion:**

Anterior vaginectomy, despite our best attempts, is perhaps not sufficient to prevent vaginal cuff recurrence. Therefore, follow-up evaluation is essential, and further studies are necessary to address the optimal approach for initial management.

**Patient Summary:**

Although vaginal cuff recurrence is an unusual site of recurrence, careful evaluation is needed before cystectomy and during follow-up to identify patients at risk.

## INTRODUCTION

Radical cystectomy with pelvic lymph node dissection is the gold standard for both muscle invasive bladder cancer, and high-risk non-muscle invasive bladder cancer (1). Female patients with ag-gressive disease can be treated with anterior pelvic exenteration, which includes cystectomy, bilateral pelvic lymph node dissection, hysterectomy, bilateral salpingo-oophorectomy, and resection of the upper third of the anterior vaginal wall (2). Since local tumor extension may affect reproductive organs, anterior pelvic exenteration aims to achieve a balance between local cancer control and adequate sexual outcomes. However, it is not clear when the routine removal of reproductive organs is required to avoid local recurrence (3, 4). Recurrence after radical cystectomy is not unusual, with estimated 5-year recurrence rates of 30%-52% and cancer-specific mortality rates of 28%-35% (5–9).

The incidence of local invasion involving gynecological organs at the time of radical cystectomy ranges from between 2.7% to 7.5% in patients undergoing anterior pelvic exenteration (10–12). The vagina is the sexual organ most frequently found involved by bladder cancer, with an estimated frequency of 4.8% (13). However, vaginal cuff recurrence following radical cystectomy has never been specifically analyzed. A better understanding of this particular site of recurrence would indeed optimize female patient selection, counseling for reproductive organ preservation, and indication for orthotopic neobladder, with the purpose of improving quality of life and preserving sexual health. In response to these needs, our primary aim is to assess the inci-dence, overall survival, and cancer-specific survival rates for patients with vaginal cuff recurrence following radical cystectomy. The secondary aim is to identify possible independent predictors of bladder cancer recurrence in the vaginal cuff.

## MATERIALS AND METHODS

Following institutional board review approval, a retrospective cohort study was conducted at Mayo Clinic. A prospectively maintained institutional bladder cancer registry was queried to identify women who underwent radical cystectomy for curative intent between 1985 and 2012, but had no evidence of metastatic bladder cancer. Exclusion criteria included known metastatic disease at surgery, incomplete follow-up data, and failure to provide research consent. Cystectomy usually involved anterior pelvic exenteration with removal of the bladder, cervix, uterus, and anterior vaginal wall. Anterior vaginectomy, defined as the removal of the anterior vaginal wall at the time of radical cystectomy, was performed in the presence of aggressive cancer with evidence of extravesical extension (cT3 and cT4 disease). Patients with a clinically resectable bladder tumor not involving the bladder neck, were considered candidates for an orthotopic urinary diversion. In order to achieve proper placement and support of neobladder, a urethral and vaginal sparing technique was performed. In all other cases, the urethra and external ostium of the uterus were removed. Sacrocolpopexy was not included as part of the radical cystectomy.

Owing to the retrospective nature of this study, post-operative surveillance after radical cystectomy was not standardized. However, at our institution, patients are typically followed every 3 months for the first two years after surgery, every 6 months for the subsequent two years, and then annually thereafter. Oncologic surveillance included history, physical examination, urine cytology, and radiologic imaging of the chest, abdomen, and pelvis.

**Figure 1:**
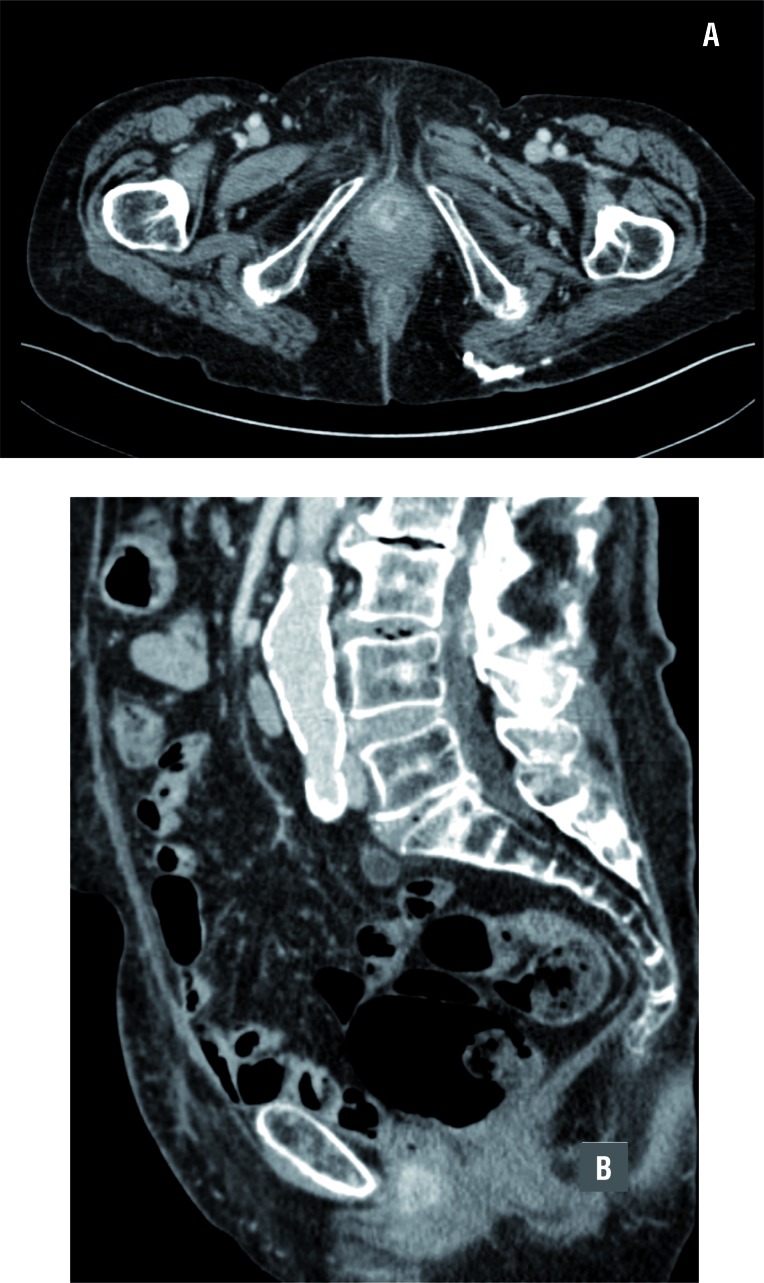
Vaginal cuff recurrence in an 82 year old female. After undergoing radical cystectomy and lymphadenectomy (pT3aN0 tumor), patient subsequently developed symptomatic vaginal blood spotting. The CT scan demonstrates a solid mass originating from the vaginal cuff. Surgery was performed and final pathology showed a recurrence of urothelial carcinoma originating from the vaginal cuff.

Cancer recurrence was defined as evidence of bladder cancer on imaging after radical cys-tectomy (Figure-1), with or without the presence of symptoms. Radiographic studies included bone scintigraphy, positron emission tomography/computed tomography scan, computed tomography and magnetic resonance imaging. Biopsy confirmation of bladder cancer recurrence was performed in the presence of considerable diagnostic uncertainty. Clinical recurrences were categorized as either vaginal recurrence, or recurrence at another local, pelvic, or distant site. Vaginal cuff recurrence was defined as radiographic recurrence in the vaginal cul-de-sac invading the posterior vagina.

The date of recurrence was defined as the date of first positive imaging or positive biopsy for bladder cancer recurrence. Last follow-up date was the last date of visit or correspondence between the patient and the institution. Causes of death were identified from death certificates and physician correspondence. Bladder cancer was considered the cause of death when it was listed as the main or first cause. Data regarding administration of perioperative chemotherapy and radiation were also collected.

Frequencies and proportions were used to summarize categorical variables. Means, medians, and interquartile ranges were reported for continuously coded variables. The t test and χ^2^ test were used to compare the statistical significance of differences in mean and proportions, respectively. Kaplan-Meier analyses were performed to evaluate overall mortality and cancer specific mortality-free survival rate after radical cystectomy for patients with vaginal cuff recurrence, patients with cancer recurrence at another site, and those without recurrence. Log-rank analyses were used to compare overall mortality and cancer specific mortality among the aforementioned groups. Multivariate Cox proportional hazards models were then used to assess independent predictors of vaginal cuff recurrence. The following variables were included in the multivariate model: orthotopic vs. ileal conduit, anterior vaginectomy, administration of perioperative chemotherapy, pathologic T stage (pT>2 vs. pT≤2), and bladder tumor location at radical cystectomy (anterior vs. posterior). Tumor in the posterior bladder wall, base, trigone, posterior urethra, and ureterovesical junction at radical cystectomy were described as posterior in location. All analyses were performed using the SAS statistical package (SAS Institute, Inc).

## RESULTS

The study included 469 women with bladder cancer who received treatment with radical cystectomy. Vaginal cuff recurrence was detected in 34 patients (7.3%). Recurrence at other sites was identified in 130 patients (27.7%), and no recurrence occurred in 305 patients (65%). Tables 1 and 2 describe the baseline characteristics of patients with vaginal cuff recurrence, other sites of cancer recurrence and those without recurrence. The three study groups did not demonstrate significant differences with regards to the analyzed variables.

**Table 1:** Distribution of Risk Factors Among Vaginal Cuff Recurrence (A) vs. Recurrence at Other Sites (B) vs. No Recurrence (C).

		Vaginal Cuff Recurrence	Recurrence at Other Sites	No Recurrence	*p* Value	*p* Value
		(n=34)	(n=130)	(n=305)	A vs B	A vs C
		(A)	(B)	(C)		
**Age**					0.2	0.5
	Median(IQR)	70.5 (63-76)	68 (62-74)	69 (62-76)		
**Decade**					0.3	0.3
	1980s	8 (23%)	37 (28%)	75 (25%)		
	1990s	18 (53%)	50 (38%)	121 (40%)		
	2000s	8 (23%)	43 (33%)	109 (36%)		
**ECOG performance status**					0.7	0.8
	0	24 (71%)	98 (75%)	222 (73%)		
	1	8 (23%)	22 (17%)	56 (18%)		
	2	2 (6%)	8 (6%)	22 (7%)		
	3	0	2 (1%)	5 (2%)		
**BMI**					0.5	0.2
	BMI ≤25	12 (35%)	63 (48%)	149 (49%)		
	BMI 26-30	14 (41%)	40 (31%)	100 (33%)		
	BMI 31-35	7 (21%)	21 (16%)	36 (12%)		
	BMI >35	1 (3%)	6 (5%)	20 (7%)		
	Orthotopic urinary diversion	5 (15%)	20 (15%)	47 (15%)	0.9	0.9
	**pT**					
	≤pT2	18 (53%)	58 (45%)	189 (62%)	0.5	0.6
	pT>2	16 (47%)	72 (55%)	116 (38%)		
**pN**					0.4	0.3
	NX	2 (6%)	20 (15%)	50 (16%)		
	N0	28 (82%)	87 (67%)	223 (73%)		
	N1	1 (3%)	12 (9%)	15 (5%)		
	N2	3 (9%)	10 (8%)	13 (4%)		
	N3	0	1 (1%)	4 (1%)		
**Lymph nodes at RC**						
	Positive	4 (12%)	23 (18%)	32 (10%)	0.4	0.8
Peripheral tumor margin		0	4 (3%)	5 (2%)	0.3	0.7
Perioperative chemotherapy		7 (21%)	21 (16%)	32 (10%)	0.5	0.1
Median Time to last follow-up among alive in years (IQR)		6.3 (6-19)	12.5 (9-26)	12.9 (8-17)	0.4	0.4
Smoker		19 (56%)	75 (58%)	178 (58%)	0.8	0.8
Prior pelvic radiation		1 (3%)	7 (5%)	178 (58%)	0.6	0.5
Multifocal tumor		0	11 (8.5%)	13 (4%)	0.1	0.2

**Table 2:** Distribution of Risk Factors Among Vaginal Cuff Recurrence vs No Recurrence.

		No Recurrence	Vaginal Cuff Recurrence	Total	*P* Value
		(n=305)	(n=34)	(N=339)	
**Age**					0.49
	N	305	34	339	
	Mean (SD)	68.1 (10.9)	70.0 (8.2)	68.3 (10.7)	
	Median	69	70.5	70	
	Q1, Q3	62.0, 76.0	63.0, 76.0	62.0, 76.0	
	Range	19.0-91.0	53.0-86.0	19.0-91.0	
**Decade**					0.27
	1980s	75 (24.6%)	8 (23.5%)	83 (24.5%)	
	1990s	121 (39.7%)	18 (52.9%)	139 (41.0%)	
	2000s	109 (35.7%)	8 (23.5%)	117 (34.5%)	
**ECOG performance status**					0.78
	0	222 (72.8%)	24 (70.6%)	246 (72.6%)	
	1	56 (18.4%)	8 (23.5%)	64 (18.9%)	
	2	22 (7.2%)	2 (5.9%)	24 (7.1%)	
	3	5 (1.6%)	0 (0.0%)	5 (1.5%)	
**BMI**					0.23
	Missing	1	0	1	
	BMI≤25	148 (48.7%)	12 (35.3%)	160 (47.3%)	
	BMI 26-30	100 (32.9%)	14 (41.2%)	114 (33.7%)	
	BMI 31-35	36 (11.8%)	7 (20.6%)	43 (12.7%)	
	BMI >35	20 (6.6%)	1 (2.9%)	21 (6.2%)	
**Orthotopic urinary diversion**					0.91
	Missing	1	0	1	
	0=No	257 (84.5%)	29 (85.3%)	286 (84.6%)	
	1=Yes	47 (15.5%)	5 (14.7%)	52 (15.4%)	
**pT**					0.59
	≤pT1	133 (43.6%)	13 (38.2%)	146 (43.1%)	
	pT2	56 (18.4%)	5 (14.7%)	61 (18.0%)	
	pT3/T4	116 (38.0%)	16 (47.1%)	132 (38.9%)	
**pN**					0.33
	NX	50 (16.4%)	2 (5.9%)	52 (15.3%)	
	N0	223 (73.1%)	28 (82.4%)	251 (74.0%)	
	N1	15 (4.9%)	1 (2.9%)	16 (4.7%)	
	N2	13 (4.3%)	3 (8.8%)	16 (4.7%)	
	N3	4 (1.3%)	0 (0.0%)	4 (1.2%)	
**Lymph nodes at RC**					0.82
	Negative	273 (89.5%)	30 (88.2%)	303 (89.4%)	
	Positive	32 (10.5%)	4 (11.8%)	36 (10.6%)	
**Peripheral tumor margin**					0.67
	No	298 (97.7%)	34 (100.0%)	332 (97.9%)	
	Yes	5 (1.6%)	0 (0.0%)	5 (1.5%)	
**Perioperative chemotherapy**					0.08
	No	273 (89.5%)	27 (79.4%)	300 (88.5%)	
	Yes	32 (10.5%)	7 (20.6%)	39 (11.5%)	
**Time to last follow-up**					0.38
N		108	3	111	
Mean (SD)		13.8 (7.1)	10.3 (7.5)	13.7 (7.1)	
Median		12.9	6.3	12.8	
Q1, Q3		8.1, 17.4	5.7, 19.0	7.9, 17.5	
Range		0.0-32.6	5.7-19.0	0.0-32.6	
**Smoker**					0.78
	Never	127 (41.6%)	15 (44.1%)	142 (41.9%)	
	Yes	178 (58.4%)	19 (55.9%)	197 (58.1%)	
**Prior pelvic radiation**					0.48
	No	287 (94.1%)	33 (97.1%)	320 (94.4%)	
	Yes	18 (5.9%)	1 (2.9%)	19 (5.6%)	
**Multifocal**					0.22
	No	292 (95.7%)	34 (100.0%)	326 (96.2%)	
	Yes	13 (4.3%)	0 (0.0%)	13 (3.8%)	

**Figure 2:**
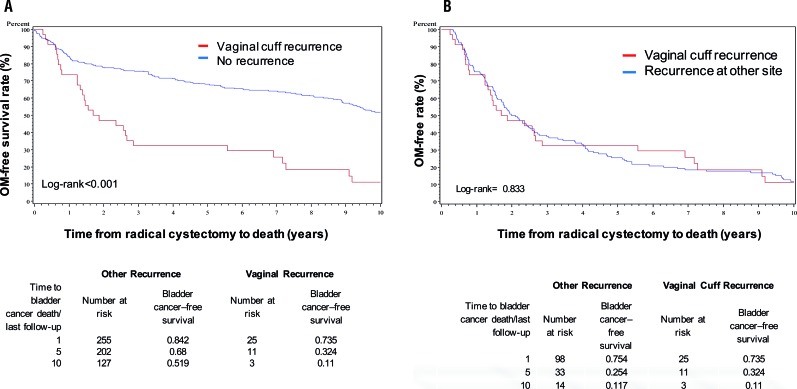
Overall Mortality (OM) for vaginal cuff Recurrence vs No Recurrence (2a) and vs Recurrence at Other Site (2b).

**Figure 3:**
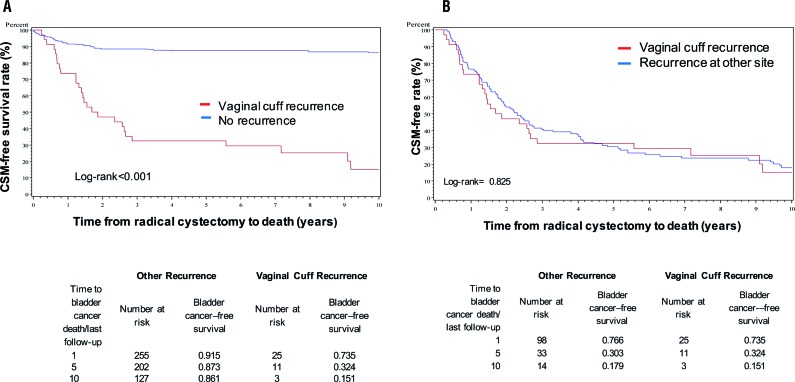
Cancer Specific Mortality (CSM) for Vaginal Cuff Recurrence (3a) vs No Recurrence and vs Recurrence at Other Site (3b).

A significant difference was demonstrated when comparing overall mortality and cancer specific mortality-free rate between patients with vaginal cuff recurrence and patients without any re-currence (log-rank <0.001) (Figures 2a and 3a). The 5-year overall survival rate was 32.4% for patients with vaginal cuff recurrence and 25.0% for other sites of recurrence (Figure-2b). Cancer-specific survival rate was 32.4% for vaginal cuff recurrence and 30.3% for other site of recurrence (Figure-3b). No significant differences were found when comparing overall mortality and cancer specific mortality-free rates between those with vaginal cuff recurrence, and patients with recurrence at other sites (Log-Rank p=0.83 and 0.82, respectively).

When univariate and multivariate Cox proportional hazards regression analysis compared vaginal cuff recurrence to recurrences at other sites, the presence of bladder cancer in the posterior floor was found to be independently associated with a lower risk of vaginal cuff recurrence (Ha-zard Ratio [HR], 0.346 [95% CI, 0.159-0.0104]; P=0.007) (Table-3). On multivariate analysis, an-terior vaginectomy was independently associated with development of vaginal cuff recurrence (HR, 2.595 [95% CI 1.077-6.249]; P=0.03) (Table-3).

**Table 3:** Multivariate Association of Risk Factors with Vaginal Cuff Recurrence vs Recurrence at Other Site.

Variables		Univariate				Multivariate			
		HR estimate	95% CI for OR		P value	HR estimate	95% CI for OR		P value
**Localization**									0.01
	Posterior	Ref.			0.01	Ref.			
	Anterior	0.346	0.159	0.751		0.353	0.159	0.783	
**Diversion**									0.99
	Incontinent	Ref.				Ref.			
	Continent	1.117	0.388	3.218	0.84	0.991	0.303	3.242	
**Vaginectomy**									0.03
	No	Ref.			0.07	Ref.			
	Yes	2.011	0.934	4.329		2.595	1.077	6.249	
**Perioperative chemotherapy**									0.28
	Yes	Ref.				Ref.			
	No	1.346	0.519	3.492	0.54	1.811	0.620	5.295	
pT2 vs ≤pT1		0.629	0.197	2.007	0.68	0.587	0.170	2.030	0.80
pT3/T4 vs ≤pT1		0.615	0.267	1.417	0.53	0.460	0.181	1.172	0.26

**CI** = confidence interval; **OR** = odds ratio

Treatment for recurrences were as follows: five (14.7%) patients underwent salvage surgery, 20 (76.4%) received adjuvant chemotherapy, and three (8.9%) patients were treated with palliative radiation therapy.

## DISCUSSION

Recurrence patterns of disease among women following radical cystectomy have been poorly described in the literature. Current guidelines marginally address the issue of sexual-sparing cystectomies in the female population. Women have been reported to experience worse cancer outcomes than men with locally advanced bladder tumors (14, 15). The etiology for this difference in outcome remains to be established, but is presumed to result from delayed presentation of disease, healthcare disparities (16), vesical anatomy, and different levels of sex hormones (17). The largest studies have defined recurrent bladder urothelial carcinoma as the presence of recurrent cancer within the soft tissue field of surgical resection, without particular attention to the differences in pelvic anatomy between males and females (15, 18–20). We believe, however, that a more precise study is crucial for treatment, prognosis and follow-up purposes.

In this context, we found a 7.3% incidence of vaginal cuff recurrence among women following radical cystectomy. In the current literature, a limited number of studies evaluate the involvement of reproductive organs at final radical cystectomy pathology. In particular, Ali-el-Dein et al. (12) estimated secondary malignant involvement of reproductive organs in female cystectomy spe-cimens in 2.6% of cases. In another study, the vagina has been described as the most commonly involved reproductive organ at radical cystectomy, with an incidence of 4.8% (13). Our findings demonstrate a significant difference in both overall mortality and cancer specific mortality-free survival between patients with vaginal cuff recurrence and patients without recurrence. Interestingly, no survival differences were found between vaginal cuff recurrence and recurrence at other sites. Since vaginal cuff recurrence is not an uncommon site of recurrence, vaginal examination should be recommended during routine follow-up post radical cystectomy, and any abnormal vaginal secretion or bleeding further investigated. Tumor position involving the posterior bladder, and surgery with anterior vaginectomy were statistically significant independent predictors of vaginal cuff recurrence at multivariate analysis. The anterior vaginal wall was removed to achieve negative surgical margins and to prevent local recurrence. Surprisingly, tumor stage did not predict vaginal cuff recurrence. However, anterior vaginectomy, performed in a surgical attempt to avoid local recurrence, may be seen in our study as a marker of increased risk for recurrence in the cul-de sac or in the remaining vaginal canal.

Although anterior vaginectomy is perhaps not good enough to prevent recurrence, there are no current guidelines regarding the optimal management of patients at increased risk of recurrence. These results are interesting for several reasons. First, tumor position and multifocality in the bladder must be evaluated in the preoperative setting. Adequate bladder sampling during transurethral resection and bimanual examination under anesthesia are both of great importance. A careful evaluation of preoperative imaging is also necessary before a reproductive organ sparing approach. Diffusion-weighted magnetic resonance imaging may be considered the technique of choice for local staging, as there is increasing evidence of its superior performance compared to other imaging techniques (21, 22). Second, vaginal sparing surgery should be suggested only to a select group of patients. Even if vaginal preservation has potential benefits to decrease postoperative rates of sexual dysfunction, decreased risk of pouch prolapse, and neobladder-vaginal fistula, vaginal cuff recurrence has a poor 5-year cancer-specific survival. Therefore, patient characteristics (e.g., age, performance status), patient needs (e.g., sexual desire, choice of diversion), pathological stage, and position of tumor in the bladder (organ-confined tumors away from the bladder neck, trigone, bladder base, and posterior wall) should be carefully evaluated. Any residual or recurrent disease in the pelvis is a poor predictor of overall survival and cancer-specific survival and adds to the body of literature supporting the need for meticulous extirpative surgery at the time of radical cystectomy.

The present study evaluated vaginal cuff recurrence as a specific site of relapse. This particular attention to a unique site of recurrence in the female population could explain the detection of a relatively high frequency of vaginal cuff recurrence in comparison to other published series (12, 13). Due to the well-known differences in pelvic anatomies between males and females, our results stressed the importance of gender differences in the evaluation of recurrence. Even though further studies are needed, these results add value for improved comprehension and possible prevention of bladder cancer recurrence.

However, several limitations of the study include small sample size and the small number of events which limited further statistical sub-analysis. Additionally, due to the retrospective nature of the study, certain biases may be present in the analysis. Examples include the advances in treatment and imaging technologies, different operating surgeons performing radical cystectomy, and the differences in follow-up schedules. All of these variables could not be appropriately controlled for and may have impacted outcomes.

## CONCLUSIONS

Assessing the incidence and potential risk factors for development of recurrent disease among women with bladder cancer, demonstrates that vaginal cuff recurrence is associated with similar survival outcomes to recurrence occurring at other locations. The selection of appropriate candidates for a sexual-sparing procedure must carefully balance both organ preservation and oncologic control. Furthermore, careful evaluation of the location of bladder cancer prior to surgery is crucial for appropriate surgical planning. Additional studies are required to better evaluate the impact of vaginal cuff recurrence on survivorship and the appropriate management following radical cystectomy.

## References

[1] Witjes JA, Compérat E, Cowan NC, De Santis M, Gakis G, Lebret T (2014). EAU guidelines on muscle-invasive and metastatic bladder cancer: summary of the 2013 guidelines. Eur Urol..

[2] Marshall FF, Treiger BF (1991). Radical cystectomy (anterior exenteration) in the female patient. Urol Clin North Am..

[3] Koie T, Hatakeyama S, Yoneyama T, Hashimoto Y, Kamimura N, Ohyama C (2010). Uterus-, fallopian tube-, ovary-, and vagina-sparing cystectomy followed by U-shaped ileal neobladder construction for female bladder cancer patients: oncological and functional outcomes. Urology..

[4] Chang SS, Cole E, Cookson MS, Peterson M, Smith JA (2002). Preservation of the anterior vaginal wall during female radical cystectomy with orthotopic urinary diversion: technique and results. J Urol..

[5] Ploussard G, Shariat SF, Dragomir A, Kluth LA, Xylinas E, Masson-Lecomte A (2014). Conditional survival after radical cystectomy for bladder cancer: evidence for a patient changing risk profile over time. Eur Urol..

[6] Hautmann RE, de Petriconi RC, Pfeiffer C, Volkmer BG (2012). Radical cystectomy for urothelial carcinoma of the bladder without neoadjuvant or adjuvant therapy: long-term results in 1100 patients. Eur Urol..

[7] Stein JP, Lieskovsky G, Cote R, Groshen S, Feng AC, Boyd S (2001). Radical cystectomy in the treatment of invasive bladder cancer: long-term results in 1,054 patients. J Clin Oncol..

[8] Yafi FA, Aprikian AG, Chin JL, Fradet Y, Izawa J, Estey E (2011). Contemporary outcomes of 2287 patients with bladder cancer who were treated with radical cystectomy: a Canadian multicentre experience. BJU Int..

[9] Nuhn P, May M, Sun M, Fritsche HM, Brookman-May S, Buchner A (2012). External validation of postoperative nomograms for prediction of all-cause mortality, cancer-specific mortality, and recurrence in patients with urothelial carcinoma of the bladder. Eur Urol..

[10] Chen ME, Pisters LL, Malpica A, Pettaway CA, Dinney CP (1997). Risk of urethral, vaginal and cervical involvement in patients undergoing radical cystectomy for bladder cancer: results of a contemporary cystectomy series from M.D. Anderson Cancer Center. J Urol..

[11] Chang SS, Cole E, Smith JA, Cookson MS (2002). Pathological findings of gynecologic organs obtained at female radical cystectomy. J Urol..

[12] Ali-El-Dein B, Abdel-Latif M, Mosbah A, Eraky I, Shaaban AA, Taha NM (2004). Secondary malignant involvement of gynecologic organs in radical cystectomy specimens in women: is it mandatory to remove these organs routinely?. J Urol..

[13] Djaladat H, Bruins HM, Miranda G, Cai J, Skinner EC, Daneshmand S (2012). Reproductive organ involvement in female patients undergoing radical cystectomy for urothelial bladder cancer. J Urol..

[14] Mungan NA, Aben KK, Schoenberg MP, Visser O, Coebergh JW, Witjes JA (2000). Gender differences in stage-adjusted bladder cancer survival. Urology..

[15] Chamie K, Litwin MS, Bassett JC, Daskivich TJ, Lai J, Hanley JM (2013). Recurrence of high-risk bladder cancer: a population-based analysis. Cancer..

[16] Scosyrev E, Noyes K, Feng C, Messing E (2009). Sex and racial differences in bladder cancer presentation and mortality in the US. Cancer..

[17] Lucca I, Fajkovic H, Klatte T (2014). Sex steroids and gender differences in nonmuscle invasive bladder cancer. Curr Opin Urol..

[18] Stein JP, Penson DF, Lee C, Cai J, Miranda G, Skinner DG (2009). Long-term oncological outcomes in women undergoing radical cystectomy and orthotopic diversion for bladder cancer. J Urol..

[19] Shariat SF, Karakiewicz PI, Palapattu GS, Lotan Y, Rogers CG, Amiel GE (2006). Outcomes of radical cystectomy for transitional cell carcinoma of the bladder: a contemporary series from the Bladder Cancer Research Consortium. J Urol..

[20] Kluth LA, Black PC, Bochner BH, Catto J, Lerner SP, Stenzl A (2015). Prognostic and Prediction Tools in Bladder Cancer: A Comprehensive Review of the Literature. Eur Urol..

[21] Yoshida S, Koga F, Kobayashi S, Tanaka H, Satoh S, Fujii Y (2014). Diffusion-weighted magnetic resonance imaging in management of bladder cancer, particularly with multimodal bladder-sparing strategy. World J Radiol..

[22] Hafeez S, Huddart R (2013). Advances in bladder cancer imaging. BMC Med..

